# Thirteen-Year Sequelae of Marburg Virus Disease Survival: Persistent Cardiometabolic, Immunometabolic, and Haematological Alterations in the Absence of Psychological Morbidity

**DOI:** 10.3390/pathogens14070678

**Published:** 2025-07-09

**Authors:** Jennifer Serwanga, Raymond Ernest Kaweesa, Joseph Katende Ssebwana, Goeffrey Odoch, Raymond Reuel Wayesu, Anne Daphine Ntabadde, Deborah Mukisa, Peter Ejou, FiloStudy Team, Julius Julian Lutwama, Pontiano Kaleebu

**Affiliations:** 1 Uganda Virus Research Institute (UVRI), Entebbe P.O. Box 49, Ugandajoseph.ssebwanakatende@mrcuganda.org (J.K.S.); deborah.mukisa@mrcuganda.org (D.M.); pontiano.kaleebu@mrcuganda.org (P.K.); 2 MRC/UVRI & LSHTM Uganda Research Unit, Entebbe P.O. Box 49, Uganda; peter.ejou@mrcuganda.org

**Keywords:** Marburg virus disease, long-term sequelae, cardiometabolic risk, survivors, Uganda, filovirus, haematological recovery, psychological outcomes

## Abstract

**Background**: Marburg virus disease (MVD) is a highly lethal filoviral infection, yet its long-term health consequences remain poorly understood. We present one of the most temporally distant evaluations of MVD survivors, conducted 13 years post-outbreak in Uganda, offering novel insights into chronic physiological, biochemical, haematological, and psychosocial outcomes. **Methods**: A cross-sectional, community-based study compared ten MVD survivors with nineteen age- and sex-matched unexposed controls. Clinical evaluations included vital signs, anthropometry, mental health screening, and symptom reporting. Laboratory analyses covered electrolytes, inflammatory markers, renal and liver function tests, haematology, and urinalysis. Standardised psychological assessments measured anxiety, depression, perceived stigma, and social support. **Findings**: Survivors exhibited an elevated body mass index (BMI), higher systolic and diastolic blood pressure, and lower respiratory rates compared to controls, indicating ongoing cardiometabolic and autonomic changes. These trends may reflect persistent cardiometabolic stress and potential alterations in autonomic regulation, warranting further investigation. Biochemically, survivors exhibited disruptions in serum chloride, bilirubin, and total protein levels, suggesting subclinical hepatic and renal stress. Haematological analysis revealed persistent reticulocytosis despite normal haemoglobin levels, indicating long-term erythropoietic modulation. Despite these physiological changes, survivors reported minimal psychological morbidity, sharply contrasting with the post-recovery profiles of other viral haemorrhagic fevers. Stigma was prevalent during the outbreak; however, strong family support alleviated long-term psychosocial distress. **Interpretation**: Thirteen years post-infection, MVD survivors demonstrate multisystem physiological perturbations without marked psychological sequelae. These findings challenge assumptions of universal post-viral trauma and highlight the necessity for tailored survivor care models. Future longitudinal studies should investigate the mechanistic pathways underlying cardiometabolic and haematological reprogramming to inform intervention strategies in resource-limited settings.

## 1. Introduction

Marburg virus disease (MVD) is a severe haemorrhagic illness caused by Marburg virus (MARV), a filovirus closely related to Ebola [1]. First recognised in 1967 following outbreaks in Germany and Yugoslavia linked to African green monkeys from Uganda [2,3], MARV has since caused sporadic but fatal outbreaks across Sub-Saharan Africa, including the DRC [4], Uganda [5], Guinea [6], Tanzania [7], and Rwanda [8]. In Uganda’s 2012 outbreak, 26 confirmed and probable cases were reported, with 15 deaths [5,9].

The 2012 Marburg virus disease (MVD) outbreak in Uganda marked a significant event in the region’s filovirus history due to its unique epidemiological characteristics and public health response [9]. Spanning multiple districts, the outbreak was first identified in Ibanda District and traced retrospectively to July 2012. A total of 26 confirmed and probable cases were documented, with a case–fatality rate of 58%, including 4 laboratory-confirmed fatalities. Unlike previous outbreaks, no direct zoonotic source was identified, and transmission occurred primarily through human-to-human contact, with funerals and close caregiving activities identified as key risk factors. Clinical features commonly observed in cases included hiccups, anorexia, vomiting, and dysphagia, while fatal outcomes were significantly associated with lower RT-PCR cycle threshold values, suggesting higher viral loads. The prompt institution of public health measures, including contact tracing, rapid diagnostics, the isolation of symptomatic individuals, and community sensitisation, enabled rapid containment within two weeks of recognition. This outbreak, though smaller in scale than those reported in Angola, the DRC, and recently Rwanda [10,11,12], remains one of the most clinically and virologically well-characterised MVD events in East Africa. It provides a critical reference point for survivor-based immunological studies, particularly in elucidating long-term consequences of MVD in the absence of a known zoonotic reservoir.

MARV is a zoonotic virus with Rousettus aegyptiacus bats as the probable reservoir [13]. Human infection typically arises through exposure to bats or non-human primates, followed by secondary transmission via infected bodily fluids. Symptoms emerge within 3–21 days and may progress to multiorgan failure and haemorrhage [14]. No licenced treatment exists; care remains supportive [15], though investigational therapies and vaccines are under development [16,17,18,19].

While Ebola virus disease (EVD) survivorship has been extensively studied [20,21,22,23,24,25], MVD survivor outcomes remain largely uncharacterised, despite both viruses belonging to the *Filoviridae* family and sharing similar pathogenic mechanisms, including immune evasion, vascular dysfunction, and cytokine-driven inflammation, factors known to contribute to post-viral sequelae. Ebola studies reveal long-term musculoskeletal, neurological, and psychological sequelae, along with evidence of viral persistence in immune-privileged sites [18,19]. Whether similar patterns apply to MVD is unknown. This knowledge gap limits clinical guidance and preparedness, leaving MVD survivors with unmet medical and psychosocial needs.

This study addresses that gap by systematically evaluating post-acute clinical, biochemical, and psychological outcomes among MVD survivors to inform care strategies and guide evidence-based policy.

## 2. Materials and Methods

### 2.1. Study Design and Population

We conducted a cross-sectional, community-based comparative study to evaluate long-term clinical, laboratory, and psychological sequelae in survivors of the 2012 Marburg virus disease (MVD) outbreak in Uganda. Ten adult MVD survivors and nineteen age- and sex-matched, seronegative community controls were recruited from the original outbreak catchment areas to ensure environmental comparability. Participants were enrolled at the UVRI clinic between 17 December 2024 and 25 February 2025. Survivor registries from the Ministry of Health and local health worker referrals guided recruitment. Inclusion required age ≥18 years and written informed consent; exclusions included pregnancy and acute febrile illness. Ethical approval was granted by UVRI-REC (GC/127/1045) and UNCST (HS5212ES).

### 2.2. Clinical Evaluations

Each participant underwent a structured clinical assessment by trained medical officers and certified laboratory technologists. Standardised, interviewer-administered questionnaires captured demographic variables (age, sex, education, marital status, and employment). Vital signs, including temperature, heart rate, respiratory rate, and blood pressure, were measured using calibrated automated monitors (Omron, Kyoto, Japan), in accordance with institutional SOPs. Anthropometric measurements were conducted using SECA digital stadiometers and scales, and BMIs were computed.

A complete physical examination evaluated overt clinical abnormalities. Participants provided symptom histories from acute illness through recovery using a semi-structured clinical interview. They reported the presence, severity (mild, moderate, severe), and duration (months) of key symptoms, including fatigue, myalgia, arthralgia, headache, sensory disturbances, gastrointestinal complaints, and neurological signs. Where feasible, clinical findings corroborated self-reports.

### 2.3. Clinical Laboratory Investigations

Venous blood was aseptically collected for laboratory analysis, with interpretation referenced to validated Ugandan adult intervals [26]. Complete blood counts, including leukocyte differentials, erythrocyte indices (MCV, MCHC), haemoglobin, platelet counts, and reticulocyte parameters, were obtained using Sysmex XN-Series haematology analysers. Reticulocyte volume and percentage were analysed to assess bone marrow response, particularly in survivors.

Serum biochemistry assessed electrolytes (sodium, potassium, chloride, bicarbonate), renal markers (urea, creatinine), liver enzymes (ALT, AST, ALP), total and direct bilirubin, albumin, and total protein. Glycaemic status was determined via HbA1c using high-performance liquid chromatography (HPLC), while systemic inflammation was evaluated by immunoturbidimetric quantification of C-reactive protein (CRP).

Midstream urine samples were collected in sterile containers and analysed using Siemens Multistix^®^ 10-parameter (Siemens Healthineers, Forchheim, Germany) dipsticks to detect protein, blood, glucose, ketones, bilirubin, leukocyte esterase, and nitrites. Findings were semi-quantitatively graded (negative to 3+) and interpreted for systemic or renal abnormalities.

### 2.4. Mental Health and Social Outcomes

To assess long-term psychological and social sequelae of MVD, we conducted structured mental health evaluations in all consenting survivors using validated instruments administered by a trained medical officer. In-person assessments were conducted at the UVRI clinic, more than a decade after the outbreak. Anxiety and depression were measured using the Hospital Anxiety and Depression Scale (HADS), a widely validated tool for post-infectious and chronic illness contexts. The use of the HADS questionnaire was implemented with permission from MAPI-Trust. Scores for each subscale were interpreted according to standard cut-off scores: normal levels (0–7), mild symptoms (8–10), moderate symptoms (11–15), and severe symptoms (≥16) [27]. Social factors were evaluated using binary variables that reflected experiences of stigma following recovery and the degree of support from family or the community. Instruments were interviewer-administered to eliminate literacy bias and to ensure standardisation across participants.

All responses were directly transcribed onto case report forms and double-entered into REDCap for quality control. Scoring and interpretation followed standard protocols, and no data imputation was applied. Psychological assessments were performed independently of clinical and laboratory investigations to avoid bias. No pharmacologic or counselling interventions were offered during the evaluation to preserve observational integrity.

### 2.5. Statistical Analysis

All analyses were conducted in R (v4.1.0; R Foundation for Statistical Computing, Vienna, Austria). Categorical variables were summarised as frequencies and percentages; continuous variables were described using medians with interquartile ranges (IQRs), reflecting non-normal distributions. Between-group comparisons (Marburg survivors vs. unexposed controls) employed Wilcoxon rank-sum tests for continuous variables and Fisher’s exact tests for categorical variables due to the small sample sizes. Spearman’s rank correlation coefficients (ρ) were calculated to assess the monotonic relationships between vital signs and laboratory parameters, with matrices stratified by exposure status. No multiple testing corrections were applied, given the exploratory nature. Psychological outcomes, assessed via validated scales for anxiety, depression, stigma, and social support, were scored and summarised using severity categories. Formal testing for mental health variables was not performed due to the low symptom prevalence.

## 3. Results

### 3.1. Demographic Characteristics of MVD Survivors and Controls 13 Years Post-Outbreak

**Figure 1:**
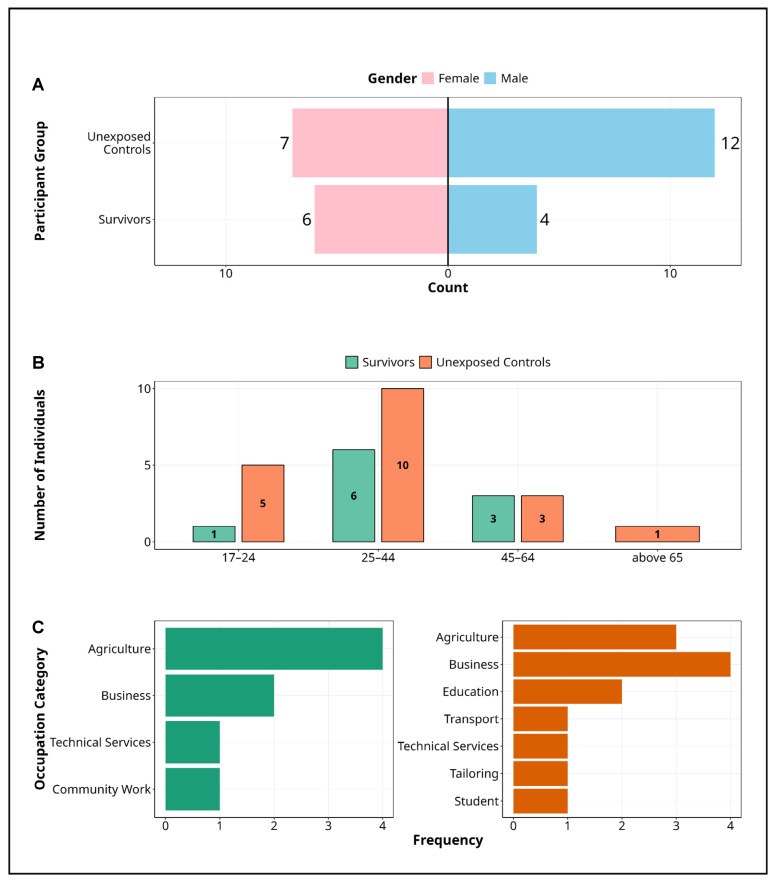
Demographic characteristics of Marburg study participants. Figure presents the demographic characteristics of study participants in the Marburg virus disease cohort, disaggregated by survivor status and unexposed controls. (**A**) Gender distribution, highlighting a higher representation of females among survivors (60%) and a predominance of males in the control group (63.2%). (**B**) Age stratification, with most participants in the 25–44 age range across both groups, although unexposed controls exhibit greater age diversity, including a larger proportion under 25. (**C**) Occupational profiles, revealing that agriculture is the primary occupation among survivors, while controls demonstrate broader diversity in business, education, transport, and technical services. These distinctions may reflect differing exposure risks and community roles during the Marburg outbreak or outcomes after recovery from the disease. **Alt Text for Figure**: Demographic characteristics of participants in the Marburg virus disease cohort, comparing survivors and unexposed controls. (**A**) There are a higher proportion of females among survivors and more males among controls. (**B**) Most participants are aged 25–44, with the unexposed controls including more individuals under 25. (**C**) Agriculture is the most common occupation among survivors, while controls are more evenly distributed across business, education, transport, and other sectors.

The study enrolled 29 participants, 10 MVD survivors (34.5%) and 19 unexposed controls (65.5%), assessed approximately 13 years after the 2012 outbreak (Figure 1, Table 1). Survivors were predominantly female (60%, *n* = 6), whereas controls were mainly male (63.2%, *n* = 12) (Figure 1A). Most participants in both groups were aged 25–44 years (survivors: 60%; controls: 52.6%), although a higher proportion of controls were under 25 (26.3% vs. 10%) (Figure 1B). Occupational profiles differed markedly: 50% of survivors were engaged in agriculture, compared to 21.1% of controls. Survivors also included individuals in technical and community roles, whereas controls showed broader diversity, including occupations in business (31.6%), education, transport, and tailoring, or being students (Figure 1C).

Socioeconomic and educational variables were captured to contextualise group characteristics. However, they were not included as covariates in the psychological outcome models, given the study’s observational design and the absence of a priori hypotheses on their predictive influence.

### 3.2. Sustained Physiological Changes in MVD Survivors

**Figure 2:**
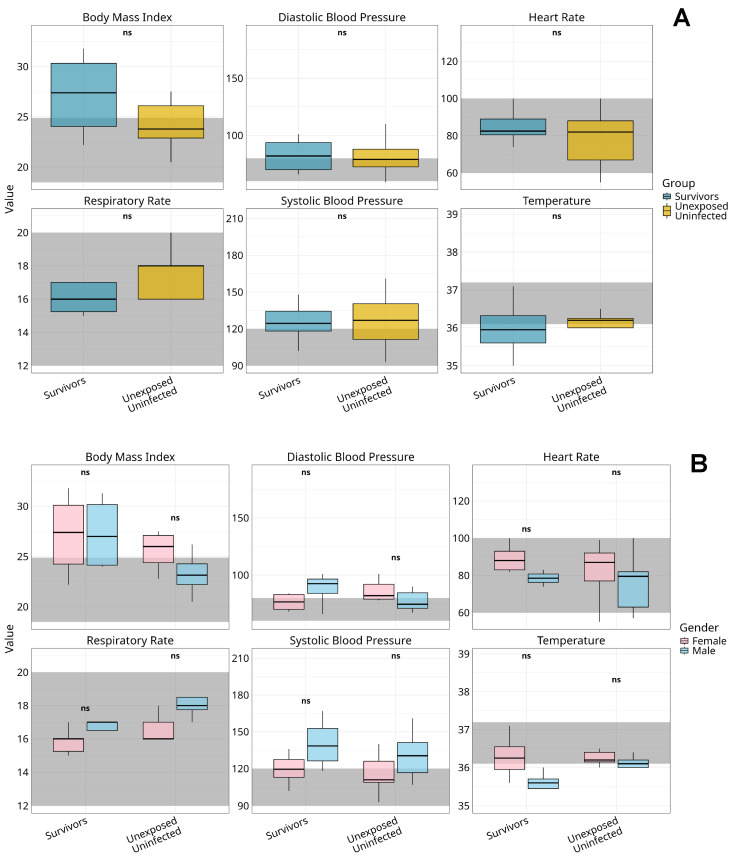
Comparative analysis of vital signs among Marburg virus survivors and unexposed controls by gender. Figure shows boxplots representing the distribution of key vital signs, body mass index (BMI), diastolic blood pressure, heart rate respiratory rate, systolic blood pressure, and temperature, across survivors and unexposed uninfected controls (**A**) and stratified by gender within two distinct groups: Marburg virus disease survivors and unexposed controls (**B**). Dark grey shaded regions indicate clinically accepted reference ranges for normal values. Median values are denoted by central lines, with interquartile ranges captured by the box boundaries. Whiskers extend to the smallest and largest non-outlier observations. Statistical analysis was performed using the Kruskal–Wallis test with Bonferroni correction for multiple comparisons; no significant differences were observed between genders across all vital sign metrics within or between groups (*p* > 0.05), as indicated by “ns” labels. The observed trends suggest physiological stability across vital parameters in both the survivor and control cohorts, with no detectable gender-specific alterations in response to Marburg virus exposure. **Alt Text for Figure:** Boxplots showing body mass index (BMI), blood pressure (systolic and diastolic), heart rate, respiratory rate, and temperature in Marburg virus disease survivors and unexposed controls. (**A**) Comparison of overall groups; (**B**) gender-stratified results. All values mostly fall within normal clinical ranges (shaded areas), with no significant gender differences observed. This indicates stable vital signs long after Marburg virus infection.

We assessed six physiological parameters, BMI, systolic and diastolic blood pressure, heart rate, respiratory rate, and body temperature, in 10 MVD survivors and 19 matched unexposed controls, 13 years post-outbreak (Figure 2).

Survivors had a higher median BMI (28.9, IQR 24.0–30.5) than controls (23.8, IQR 22.0–26.2), with most exceeding the normal range (18.5–24.9); see Figure 2A. Male survivors showed elevated systolic (129 mmHg, IQR 118–148) and diastolic (90 mmHg, IQR 66–95) blood pressure compared to female survivors (systolic: 119 mmHg, IQR 102–130; diastolic: 80 mmHg, IQR 69–84); see Figure 2B. This sex-based disparity was less evident among controls.

Median heart rates were higher in females across both groups (survivors: 89 bpm, IQR 82–95; controls: 85 bpm, IQR 76–88) than in males (survivors: 78 bpm, IQR 63–82). Respiratory rates were lower in survivors (median 16 breaths/min, IQR 15–17) than in controls (median 18, IQR 16–20). Body temperature remained stable across all groups (range 36.5–36.8 °C), with no significant sex-based or group differences.

No group differences reached statistical significance after Bonferroni-adjusted Kruskal–Wallis testing (all *p* > 0.05).

### 3.3. Narrow Symptom Spectrum in Long-Term Follow-Up of MVD Survivors

To minimise reporting bias and encourage unprompted disclosure, we employed an open-ended interview approach guided by a standardised checklist of commonly reported post-viral symptoms from prior MVD and EVD survivor studies. While the checklist included fatigue, musculoskeletal pain, neurocognitive complaints, and gastrointestinal symptoms, interviews were deliberately initiated with open-ended prompts rather than symptom-by-symptom enumeration to avoid leading responses.

**Figure 3:**
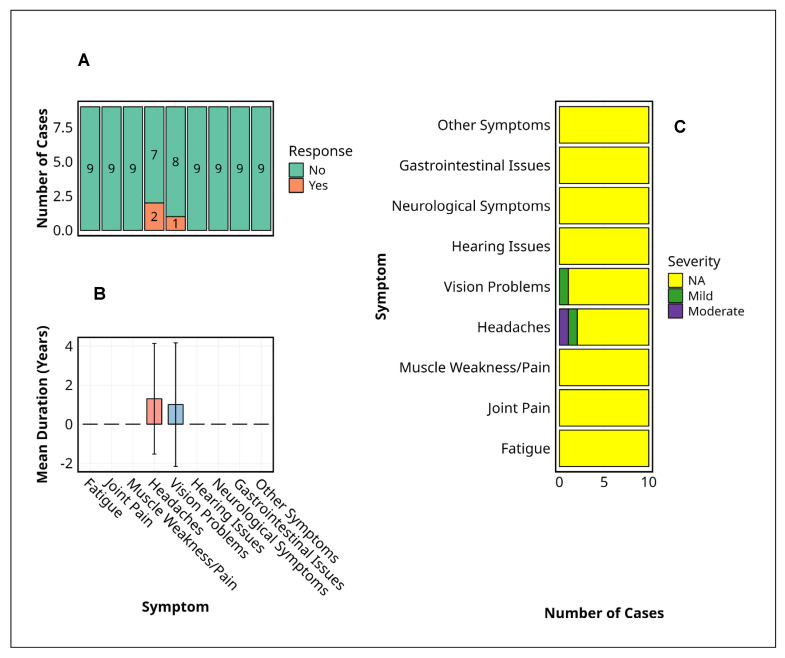
Symptom profiles among Marburg virus disease survivors. This figure summarises the prevalence, duration, and severity of persistent symptoms reported by Marburg virus disease survivors. (**A**) The prevalence of symptoms indicates the number of survivors reporting each symptom, with the majority indicating absence (“No”), while headaches and vision problems were the only symptoms with positive responses. (**B**) Mean duration of reported symptoms (years) with standard deviation represented by error bars, highlighting approximately one-year persistence for headaches and vision problems. (**C**) Symptom severity distribution was categorised as ‘Mild’, ‘Moderate’, or ‘NA’ (missing data), with ‘NA’ predominating across all symptoms, except for minor occurrences of ‘Mild’ and ‘Moderate’ severity in headaches and vision problems. **Alt Text for Figure:** Bar charts illustrating symptom profiles among Marburg virus disease survivors. (**A**) Most survivors reported no persistent symptoms; only headaches and vision problems were reported by a few individuals. (**B**) The average duration of these symptoms was approximately one year, with variability shown by error bars. (**C**) Symptom severity was mostly mild or moderate for headaches and vision problems, while other symptoms were not reported.

Among the nine MVD survivors assessed, only two chronic symptoms, headache and visual disturbance, persisted beyond the acute phase. Headaches were reported in 22% (*n* = 2) and visual problems in 11% (*n* = 1) (Figure 3A). No cases of fatigue, joint or muscle pain, hearing loss, gastrointestinal issues, or neurocognitive symptoms were recorded during follow-up.

Regarding symptom severity (Figure 3B), there was one case of moderate headache and one mild; the single visual complaint remained mild throughout. The mean duration of both persistent symptoms was approximately 12 months, with broad standard deviations indicating interindividual variability (Figure 3C). No participant reported severe symptoms, and most symptom categories were marked as not applicable (NA).

No statistical tests were applicable due to the limited symptom frequency and small sample size. Nonetheless, the restricted clinical manifestations and short persistence indicate a narrow and mild post-MVD symptom profile.

Figure 3 focuses exclusively on subjective experiences and psychosocial reflections unique to MVD survivors, capturing their lived realities in the aftermath of a life-threatening infection. Given the profound and unparalleled nature of surviving Marburg virus disease, no suitable control exists for such deeply personal and context-specific experiences. The inclusion of unexposed individuals would lack interpretive value and could risk trivialising survivor-reported outcomes. Consequently, control data are not presented in this figure.

### 3.4. Sustained Biochemical and Haematological Alterations in MVD Survivors

**Figure 4:**
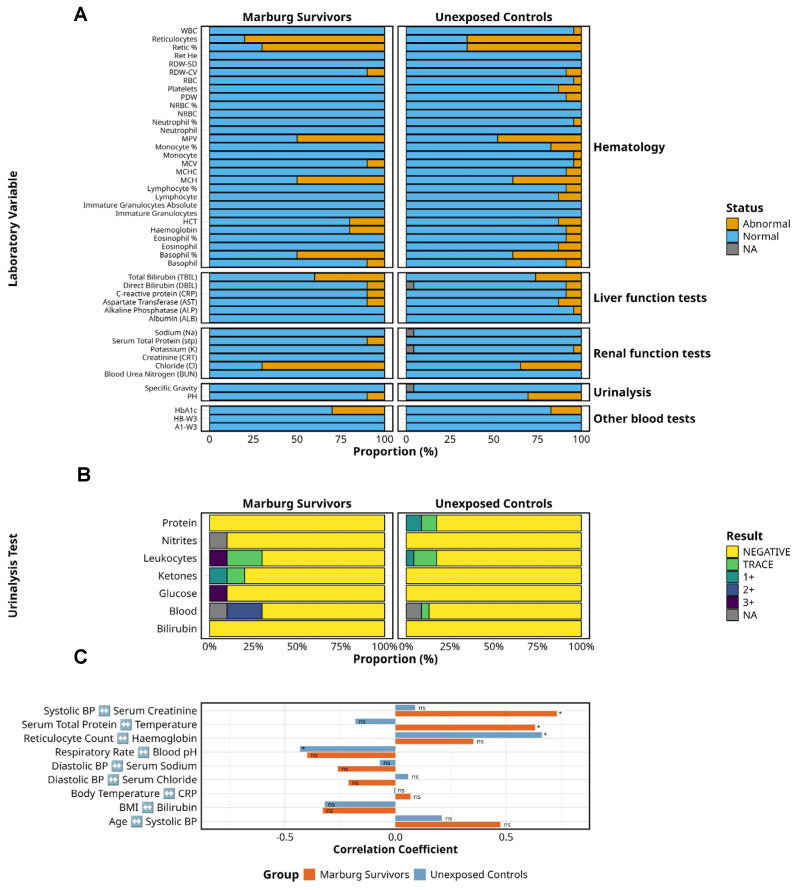
Clinical laboratory findings in Marburg survivors and unexposed controls. This figure shows laboratory and clinical parameters of Marburg virus survivors (*n* = 10) compared to unexposed controls (*n* = 19). (**A**) The distribution of normal versus abnormal laboratory findings across key test categories, including haematology, liver function, renal function, urinalysis, and other blood tests. (**B**) A detailed breakdown of urinalysis results, highlighting the proportion of negative, trace, and positive findings for key parameters. (**C**) The correlations between laboratory findings and vital signs, underscoring subtle yet persistent disruptions in electrolyte balance, liver function, haematopoietic recovery, and metabolic indicators in survivors despite apparent clinical recovery. ns = not significant. **Alt Text for Figure: Figure 4** compares laboratory and clinical findings between Marburg virus disease survivors and unexposed controls. (**A**) The number of normal and abnormal results across key test categories, including blood counts, liver and kidney function, and urinalysis. (**B**) More detail on urine test results, indicating how many participants had no abnormalities, trace levels, or positive findings. (**C**) The relationships between laboratory test results and vital signs, suggesting ongoing metabolic and organ changes in survivors despite recovery.

A comparative analysis of clinical laboratory parameters revealed persistent physiological alterations in MVD survivors (*n* = 10) relative to unexposed controls (*n* = 19) (Figure 4). Electrolyte imbalances were significantly more frequent among survivors, with elevated serum chloride observed in 70% (7/10) versus 42.1% (8/19) of controls (*p* < 0.05). Despite this, serum creatinine remained within normal limits in all survivors, indicating preserved glomerular function.

Liver function testing showed mildly elevated total bilirubin in 40% (4/10) of survivors compared to 31.6% (6/19) of controls, with no significant differences in ALT and AST levels. One survivor (10%) exhibited low total protein levels, a finding that was absent among controls. While intriguing, these abnormalities were isolated and lacked statistical power to infer pathophysiological relevance, particularly in the absence of data on malaria, alcohol intake, or hepatotoxic medications.

Haematologic indices revealed reticulocytosis in 80% (8/10) of survivors and 78.9% (15/19) of controls, with comparable reticulocyte percentages, suggesting active erythropoiesis across both groups. Other haematologic indices, including white cell counts and albumin, were similar between cohorts.

Platelet counts were within normal ranges in survivors; however, mean platelet volume abnormalities were present in both groups. HbA1c was elevated in 30% (3/10) of survivors compared to 17.4% (3/19) of controls, suggesting subclinical glycaemic impairment in a subset.

Urinalysis identified haematuria (2+) in 20% of survivors (0/19 controls), glycosuria (3+) in 10%, and ketonuria (trace–1+) in 20%, with none in controls. Leukocyte esterase was markedly elevated (3+) in 10% of survivors, with only mild or trace levels in controls. Proteinuria was absent in survivors but present in 17.4% of controls. Bilirubin and nitrites remained normal across both groups. Correlations between physiological and laboratory indices (Table 2) were exploratory and not adjusted for multiple comparisons. Apparent associations, such as a positive correlation between haemoglobin and reticulocyte counts, or an inverse relationship between respiratory rate and blood pH, likely reflect homeostatic variability rather than causal links. Similarly, the observed correlation between total serum protein and temperature lacks an established physiologic basis and may be spurious.

Overall, while this study was not powered to detect statistically significant differences, the descriptive findings highlight mild subclinical abnormalities in a subset of survivors. These warrant cautious interpretation given the small sample size and lack of matching by baseline health status. Future studies incorporating larger cohorts and longitudinal sampling, with adjustment for relevant comorbidities, are required to delineate the long-term physiological relevance of these observations.

### 3.5. Sustained Mental Health Recovery

**Figure 5:**
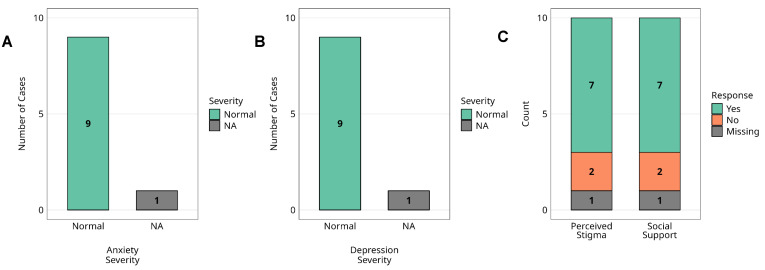
Mental health status and psychosocial factors among Marburg virus survivors. Assessment of mental health status and psychosocial factors among Marburg virus survivors (*n* = 10). (**A**) The distribution of anxiety severity levels, (**B**) depression severity levels, (**C**) perceived stigma and social support. Anxiety and depression levels are categorised based on standardised clinical measures, while perceived stigma and social support are reported through survivor self-assessment. Missing data points are indicated in each respective panel. **Alt Text for Figure:** Bar graphs showing mental health and psychosocial outcomes in 10 Marburg virus survivors. (**A**) Anxiety levels: all survivors fall within the normal range. (**B**) Depression levels, all within the normal range. (**C**) Psychosocial factors: 78% experienced stigma, and 78% reported receiving social support. One survivor had missing data for mental health assessments.

We assessed the mental health status of ten survivors of the 2012 Marburg virus disease (MVD) outbreak in Uganda, thirteen years post-infection (Figure 5). The median age at the time of illness was 23 years (IQR: 18–29), indicating that most participants were adolescents or young adults. Mental health data were completed for nine individuals.

Standardised screening tools (HADS) revealed that none of the nine survivors met the criteria for clinically significant anxiety or depressive symptoms; all scored within normal ranges. No formal psychiatric diagnoses were reported. One participant was lost to follow-up, and mental health data were unavailable.

Psychosocial assessments showed that 7/9 (77.8%) reported experiencing outbreak-related stigma, and an equal proportion (77.8%) reported receiving adequate social support at the time. One survivor, aged below five years during the outbreak and therefore not eligible for the mental health assessments, was not assessed and was included only in the perceived stigma analysis, where all ten survivors were included. No statistical comparisons were performed due to sample size limitations.

## 4. Discussion

This study provides the most temporally distant clinical and biochemical follow-up of MVD survivors to date, offering new insights into the long-term health outcomes of individuals infected during Uganda’s 2012 outbreak. We evaluated MVD survivors approximately 13 years post-infection, comparing them with unexposed community controls to assess persistent physiological, biochemical, and psychosocial sequelae. The study addresses a major knowledge gap, as post-MVD health outcomes remain largely undocumented despite an increased understanding of Ebola virus disease (EVD) survivorship [20,21,22].

Survivors exhibited sustained elevations in BMI and blood pressure, along with subtle biochemical alterations, including elevated serum chloride and bilirubin, and evidence of ongoing reticulocytosis. Despite these physiological shifts, psychological resilience was notable, with no survivors reporting clinically significant anxiety or depressive symptoms. Additionally, chronic symptoms were limited to mild, transient headaches and visual complaints in a minority of participants.

These findings fulfil the study’s primary objective: to evaluate the long-term clinical, laboratory, and psychological sequelae of MVD. The narrow symptom spectrum and the absence of psychiatric morbidity stand in contrast to the broader, multisystem impairments frequently reported in EVD survivors, including chronic pain, neurocognitive deficits, and psychological distress [28,29,30]. While our study draws comparisons to published EBOV survivor cohorts, it is important to note that most EBOV studies were conducted within significantly shorter timeframes post-infection, often less than five years [25,26,27], compared to our 13-year follow-up of MVD survivors. Consequently, direct, time-matched comparisons, particularly with respect to psychiatric outcomes, are not currently feasible.

The metabolic and vascular changes observed in this cohort, elevated BMI, and systolic/diastolic pressures are consistent with chronic cardiometabolic sequelae previously documented in EVD survivors [28]. However, the relatively mild symptom profile, limited hepatic and renal abnormalities, and lack of neurocognitive deficits differ from the EVD literature, which describes multisystem persistence, including uveitis, arthralgia, and auditory loss [21,22,23].

Psychological outcomes further diverge; whereas 47–67% of EVD survivors report clinically relevant anxiety, depression, or PTSD [29,30], none of our MVD survivors met these thresholds. This may reflect cohort-specific factors such as younger age at infection and high levels of perceived social support during convalescence, which are protective against psychiatric morbidity [30].

We hypothesise that the observed physiological alterations, particularly elevated BMI and blood pressure, may reflect chronic immune dysregulation, endothelial activation, or metabolic reprogramming induced during acute infection. We further speculate that persistent reticulocytosis despite normocytic indices could suggest ongoing marrow stimulation or subclinical haemolysis, possibly mediated by residual inflammation or altered erythropoietic signalling pathways. These interpretations remain speculative and warrant further mechanistic investigation.

Biochemical abnormalities, elevated chloride, bilirubin, and low total protein, though subclinical, may indicate latent organ stress or prior viral infection (viral legacy effects) on hepatic or renal systems. Given the absence of overt organ dysfunction, these findings may represent a compensated state of low-grade dysfunction, warranting long-term monitoring.

Importantly, the psychological resilience seen in this cohort may suggest that MVD induces a less pronounced neuroinflammatory response than EVD. Differential viral tropism, that is, the virus’s tendency to infect specific cell types or tissues, or faster immune clearance in MVD could explain the lack of neuropsychiatric sequelae and preserved cognitive outcomes.

These findings suggest that MVD survivorship is characterised more by cardiometabolic and biochemical perturbations than by multisystem or psychological morbidity. This has implications for post-epidemic care models, which currently draw heavily from EVD frameworks. Tailored strategies should emphasise cardiometabolic risk monitoring, liver and renal function screening, and community support integration, particularly in low-resource settings. The absence of psychological morbidity highlights the value of early social support interventions and may inform mental health triage strategies in future filovirus outbreaks.

A key limitation of this study is the relatively small sample size (10 survivors and 19 controls), which may reduce the statistical power and limit the generalizability of findings across the broader Sub-Saharan African context. However, this constraint reflects the high lethality of Marburg virus disease (MVD), often exceeding 80%, which has resulted in few survivors across the more than 18 documented outbreaks in the region. Many of these outbreaks were sporadic, with high case–fatality rates and limited survivor follow-up, underscoring the rarity and unique value of this survivor cohort in long-term immunological and clinical investigation [31]. Secondly, the cross-sectional design captures only a single timepoint, precluding the assessment of dynamic changes or causal inferences, and cannot clarify trajectories of recovery or progression over time. The absence of baseline (pre-infection) clinical data further limits the definitive attribution of observed abnormalities solely to MVD, as opposed to background comorbidities, environmental exposures, or ageing. Despite these constraints, the persistent alterations that we have documented, particularly in the cardiometabolic, haematological, and psychosocial domains, highlight the critical need for mechanistic investigation. To this end, we are actively leveraging retrospective and newly biobanked specimens to investigate persistent immune activation, inflammatory signatures, and viral reservoirs in immune-privileged sites. Planned studies should incorporate advanced imaging and transcriptomic and proteomic profiling to map organ-specific pathophysiology and identify durable biomarkers of disease impact. Additionally, reliance on self-reporting for symptom history introduces potential recall bias. Mental health outcomes may also be underestimated due to stigma or the limited cultural adaptation of screening tools. Given the complex psychosocial context of MVD survivorship, future research should integrate culturally adapted psychological tools and in-depth qualitative interviews to uncover lived experiences, stigma, and barriers to care.

Prospective, longitudinal studies with larger survivor cohorts are needed to validate these findings and characterise the temporal trajectory of post-MVD sequelae. The integration of imaging, immunophenotyping, and transcriptomic profiling would provide mechanistic insights into organ-specific and systemic changes. Culturally adapted, mixed-method mental health assessments are essential to ensure the comprehensive detection of psychological outcomes. Methodologically, Bayesian statistical approaches may offer superior inference in rare cohort studies and should be complemented by effect size estimates and confidence intervals to more accurately convey uncertainty and biological plausibility. Collectively, these directions will deepen our understanding of filovirus survivorship but also inform evidence-based strategies for long-term clinical management, surveillance, and vaccine-related countermeasures. This study was designed as a descriptive, observational analysis of long-term health outcomes among Marburg virus disease (MVD) survivors 13 years post-infection. While age- and sex-matched community controls are included to provide contextual reference, the data are not intended for direct causal inference and should be interpreted as exploratory and hypothesis-generating. The extended interval since acute infection and the limited sample size preclude the definitive attribution of observed differences solely to prior MARV exposure. Nevertheless, these findings provide valuable insights into possible enduring physiological and biochemical alterations following MVD survival. Plans are underway to leverage biobanked and newly collected specimens to further characterise immune and molecular biomarkers associated with disease outcomes and long-term sequelae. We recommend that future studies employ longitudinal or case–control designs with prospective sampling, the integration of acute-phase data, and multi-omics analyses to better delineate causal relationships and mechanistic pathways in post-filovirus-disease syndromes.

In conclusion, thirteen years post-infection, MVD survivors demonstrate persistent but subclinical cardiometabolic and biochemical alterations, with minimal psychological or multisystem morbidity. These findings challenge assumptions drawn from EVD survivorship and underscore the need for MVD-specific care frameworks. Targeted follow-up and survivor support are warranted to mitigate long-term health risks and ensure evidence-based post-epidemic responses.

## Figures and Tables

**Table 1:** Demographic characteristics of study participants.

Demographic Characteristics of Study Participants				
	Survivors	Unexposed and Uninfected	*p*-Value	Test
*n*	10	19		
Age (median [IQR])	38.00 [36.25, 46.75]	30.00 [24.50, 42.00]	0.260	Wilcoxon rank-sum test
Gender = Female (%)	6 (60.0)	7 (36.8)	0.270	Fisher’s exact
Gender = Male (%)	4 (40.0)	12 (63.2)	0.270	Fisher’s exact
Education (%)			0.731	Fisher’s exact
No formal education	0 (0.0)	1 (5.3)		
Primary	2 (20.0)	3 (15.8)		
Secondary	5 (50.0)	6 (31.6)		
College/university	3 (30.0)	9 (47.4)		
Employment (%)			0.309	Fisher’s exact
Employed (full-time)	3 (30.0)	5 (26.3)		
Employed (part-time)	0 (0.0)	1 (5.3)		
Self-employed	2 (20.0)	7 (36.8)		
Unemployed	4 (40.0)	1 (5.3)		
Retired	0 (0.0)	1 (5.3)		
Student	1 (10.0)	4 (21.1)		
Marital Status (%)			0.629	Fisher’s exact
Single	3 (30.0)	9 (47.4)		
Married	7 (70.0)	9 (47.4)		
Widowed	0 (0.0)	1 (5.3)		
Divorced	0 (0.0)	0 (0.0)		

**Table 2:** Comparison of Spearman’s rank correlations between Marburg survivors and unexposed controls. These correlations are exploratory observations and not adjusted for confounding.

Biologically Plausible Correlations in Marburg Disease Study				
Group	Variable Pair	Correlation (rho)	*p*-Value	Significance
Controls	Age ↔ Systolic BP	0.337	0.090	ns
Survivors	Age ↔ Systolic BP	0.470	0.210	ns
Controls	BMI ↔ Bilirubin	0.137	0.560	ns
Survivors	BMI ↔ Bilirubin	−0.330	0.320	ns
Controls	Body Temperature ↔ CRP	0.979	0.001	**
Survivors	Body Temperature ↔ CRP	0.070	0.010	ns
Controls	Diastolic BP ↔ Serum Chloride	0.755	0.010	*
Survivors	Diastolic BP ↔ Serum Chloride	−0.260	0.070	ns
Controls	Diastolic BP ↔ Serum Sodium	0.793	0.010	*
Survivors	Diastolic BP ↔ Serum Sodium	−0.210	0.060	ns
Controls	Respiratory Rate ↔ Blood pH	0.040	0.030	*
Survivors	Respiratory Rate ↔ Blood pH	−0.400	0.430	ns
Controls	Reticulocyte Count ↔ Haemoglobin	−0.001	0.980	ns
Survivors	Reticulocyte Count ↔ Haemoglobin	0.350	0.660	ns
Controls	Serum Total Protein ↔ Temperature	0.407	0.030	*
Survivors	Serum Total Protein ↔ Temperature	0.630	0.050	*
Controls	Systolic BP ↔ Serum Creatinine	0.686	0.020	*
Survivors	Systolic BP ↔ Serum Creatinine	0.730	0.090	*

Note: * indicates *p* ≤ 0.05; ** indicates *p* ≤ 0.01; ns = not significant.

## Data Availability

De-identified individual participant data underlying the findings of this study will be made available upon reasonable request to the corresponding author (jennifer.serwanga@mrcuganda.org) in accordance with the Uganda Virus Research Institute’s data access and governance policies. All requests will be reviewed for scientific merit, ethical compliance, and alignment with institutional data-sharing agreements.
